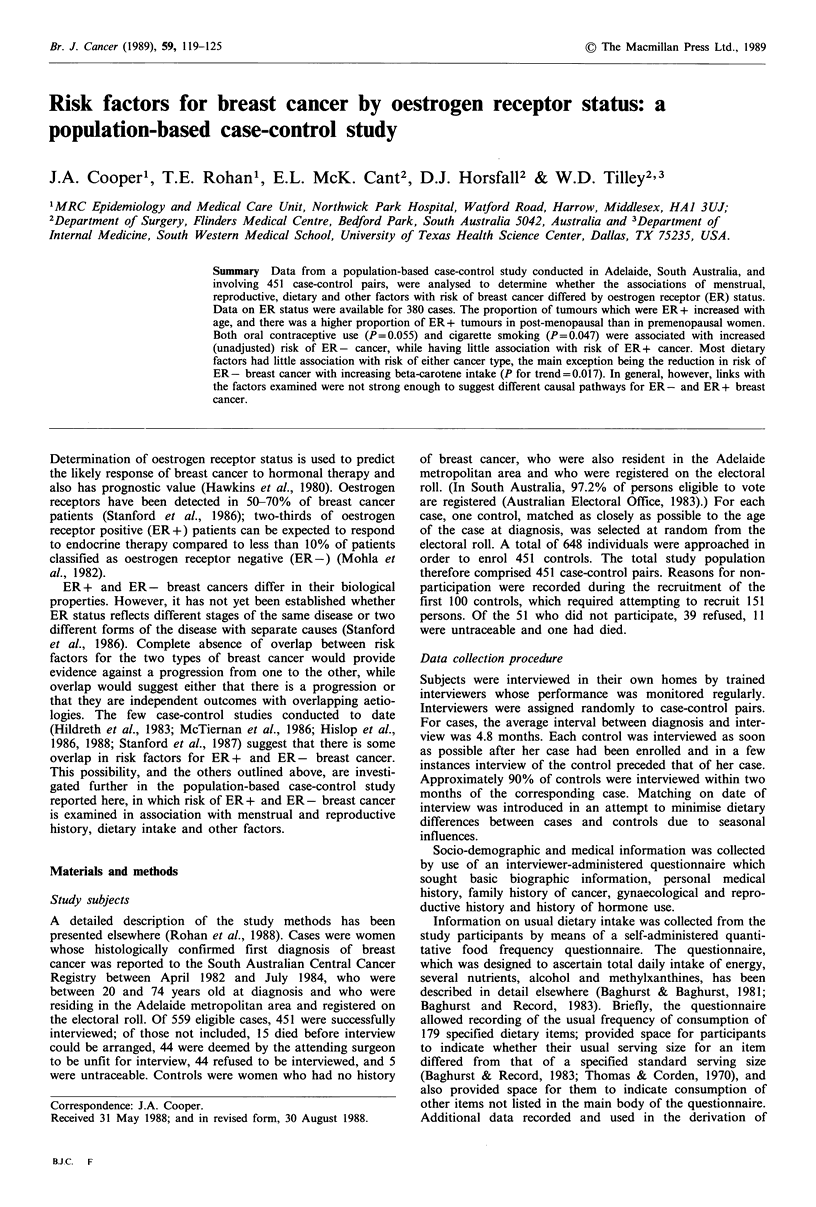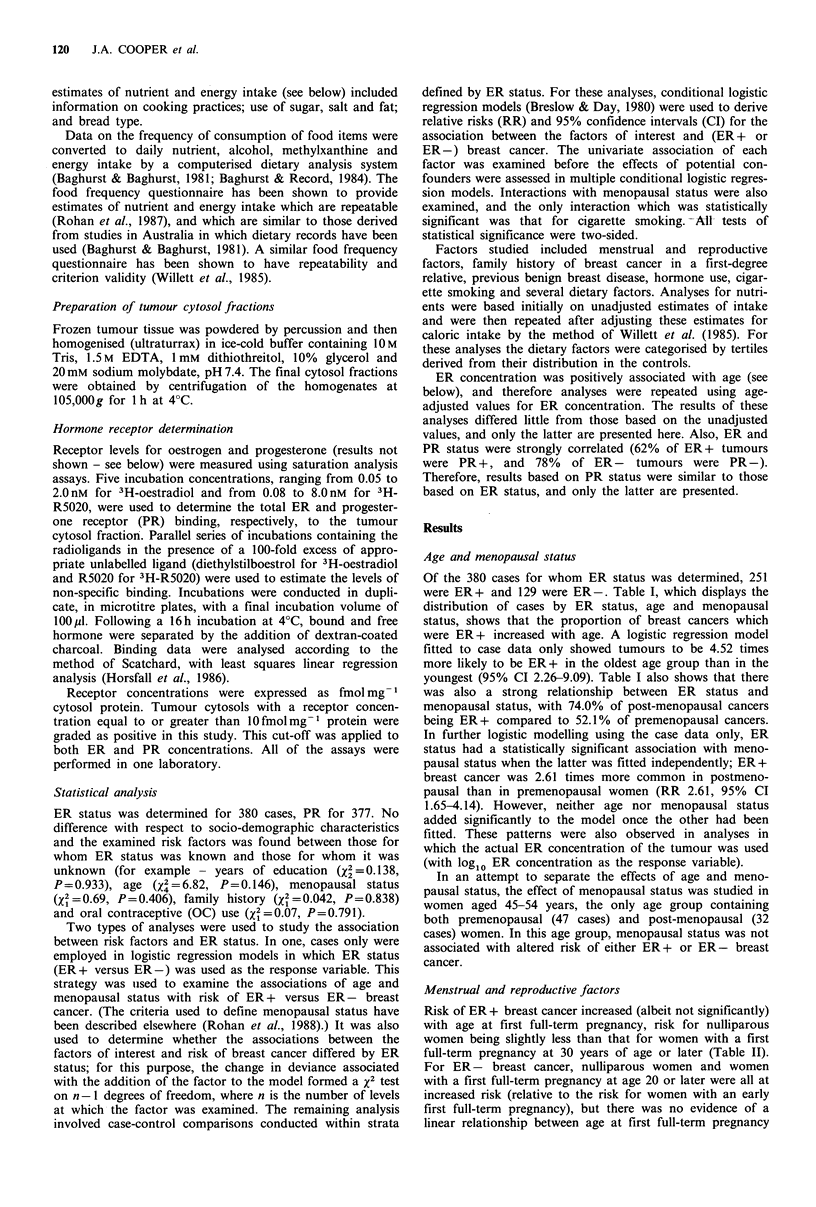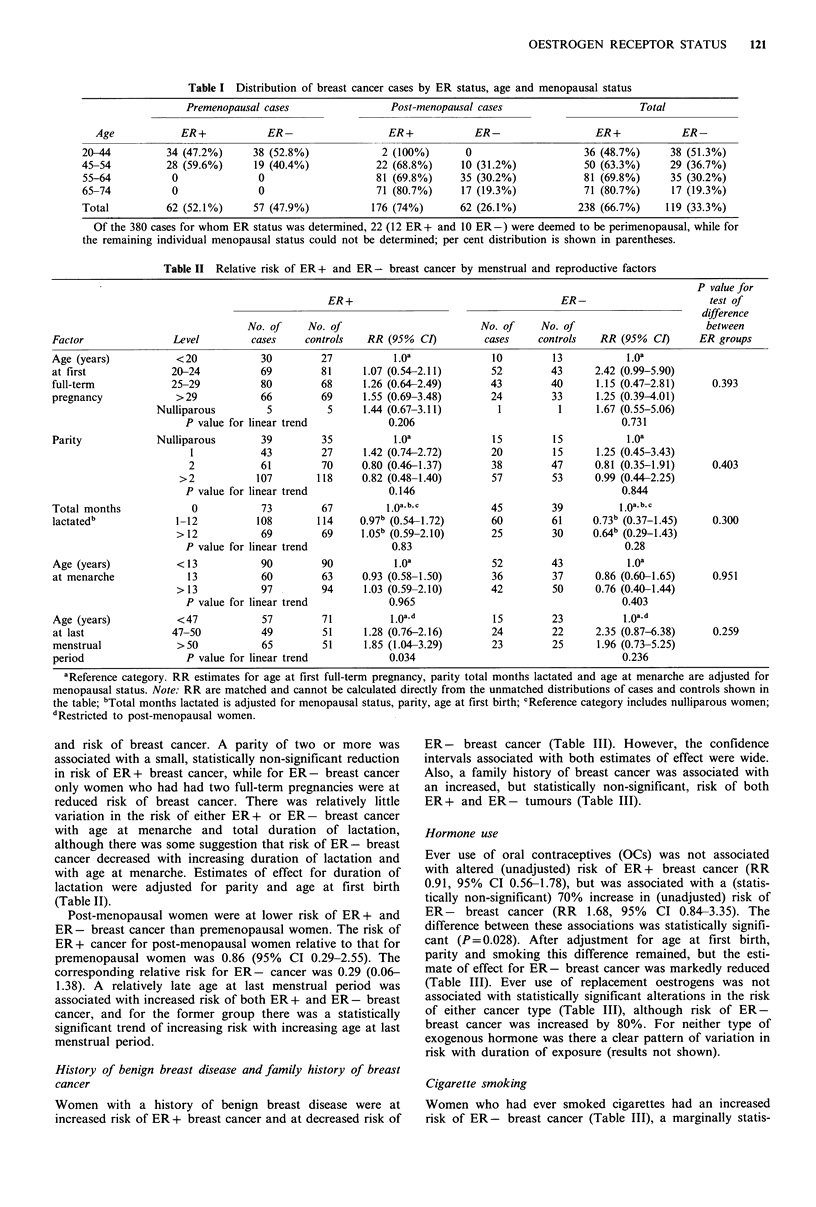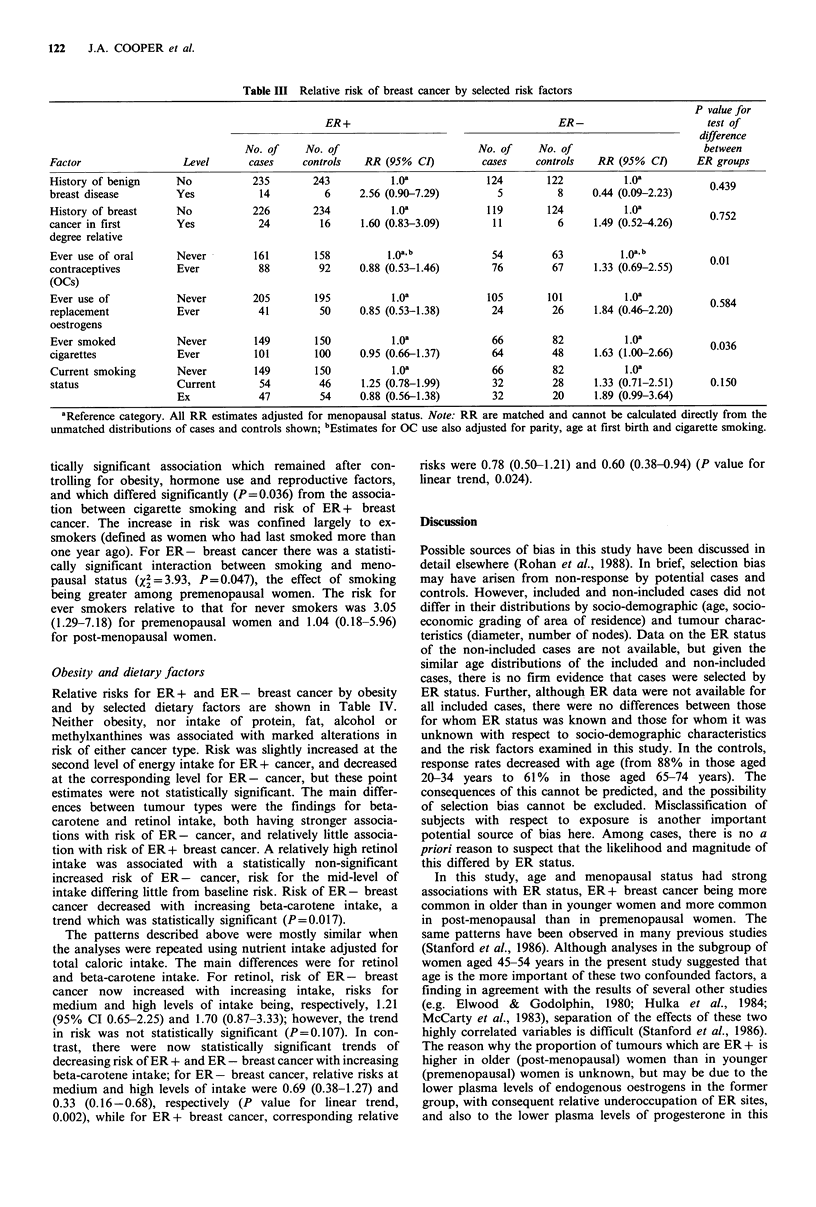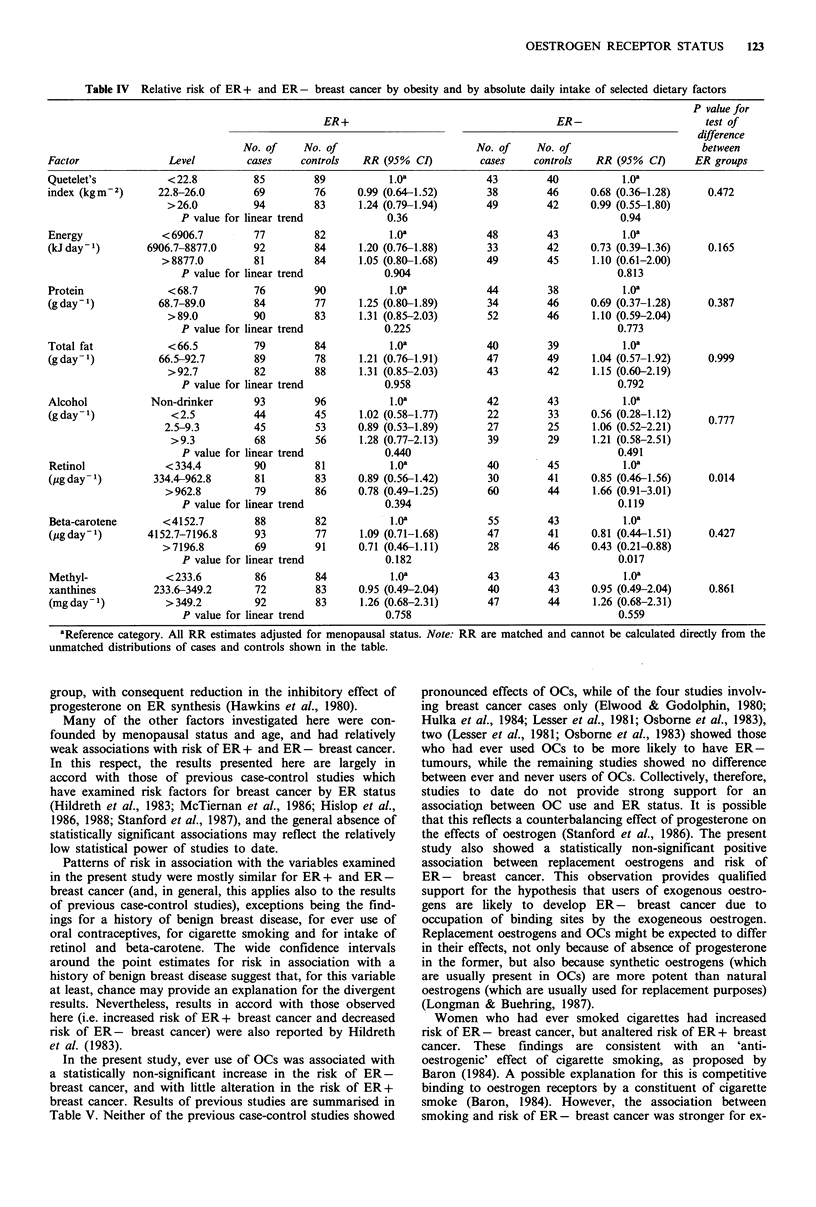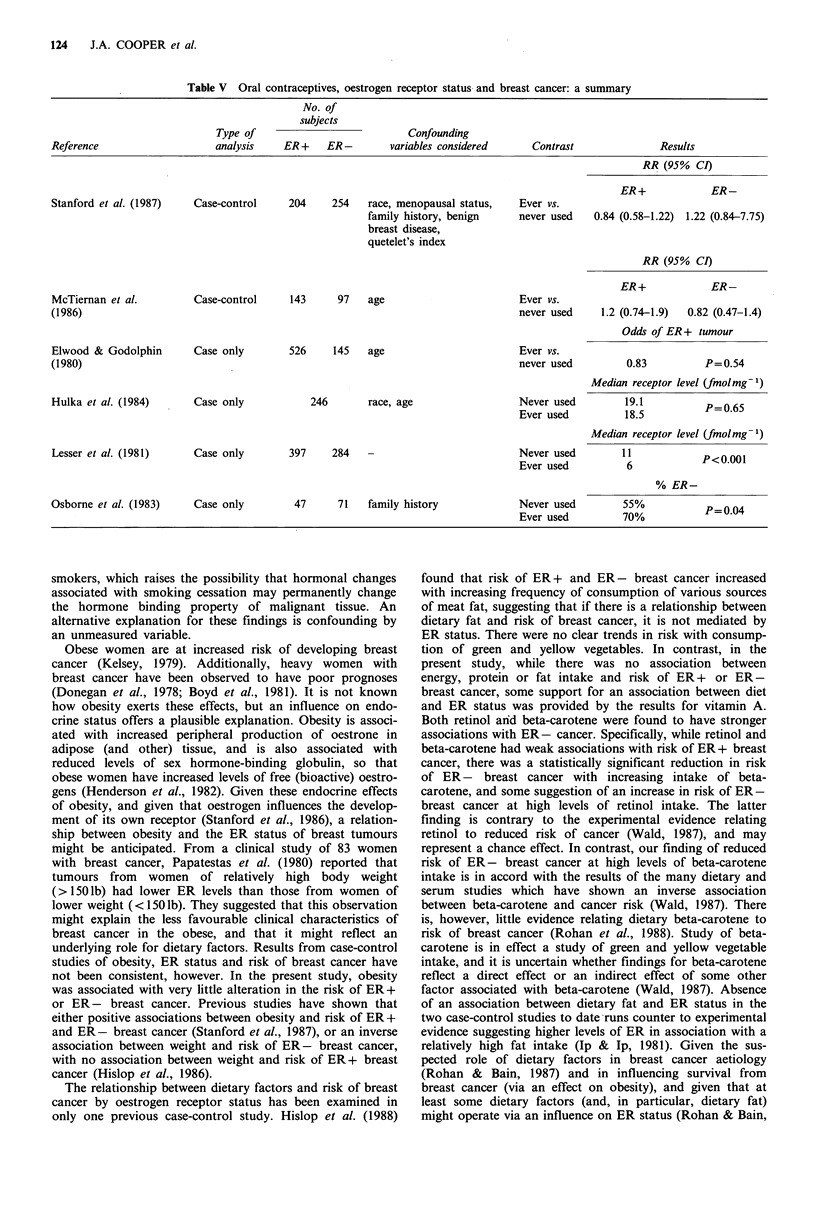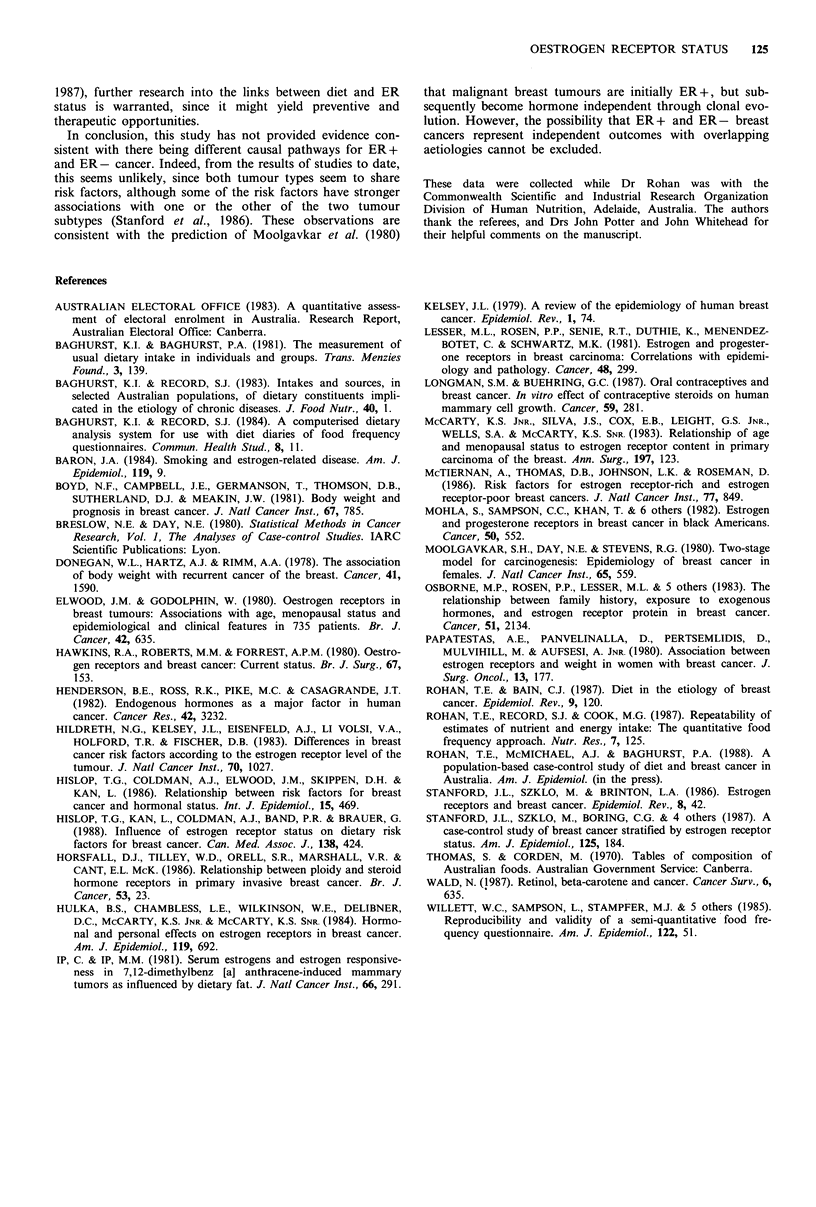# Risk factors for breast cancer by oestrogen receptor status: a population-based case-control study.

**DOI:** 10.1038/bjc.1989.24

**Published:** 1989-01

**Authors:** J. A. Cooper, T. E. Rohan, E. L. Cant, D. J. Horsfall, W. D. Tilley

**Affiliations:** MRC Epidemiology, Northwick Park Hospital, Harrow, Middlesex.

## Abstract

Data from a population-based case-control study conducted in Adelaide, South Australia, and involving 451 case-control pairs, were analysed to determine whether the associations of menstrual, reproductive, dietary and other factors with risk of breast cancer differed by oestrogen receptor (ER) status. Data on ER status were available for 380 cases. The proportion of tumours which were ER+ increased with age, and there was a higher proportion of ER+ tumours in post-menopausal than in premenopausal women. Both oral contraceptive use (P = 0.055) and cigarette smoking (P = 0.047) were associated with increased (unadjusted) risk of ER- cancer, while having little association with risk of ER+ cancer. Most dietary factors had little association with risk of either cancer type, the main exception being the reduction in risk of ER- breast cancer with increasing beta-carotene intake (P for trend = 0.017). In general, however, links with the factors examined were not strong enough to suggest different causal pathways for ER- and ER+ breast cancer.


Br. J. Cancer (1989), 59, 119–125 © The Macmillan Press Ltd., 1989

### Risk factors for breast cancer by oestrogen receptor status: a population-based case-control study

J.A. Cooper[1], T.E. Rohan[1], E.L. McK. Cant[2], D.J. Horsfall[2] & W.D. Tilley[2,3]

[1]*MRC Epidemiology and Medical Care Unit, Northwick Park Hospital, Watford Road, Harrow, Middlesex, HA1 3UJ;* [2]*Department of Surgery, Flinders Medical Centre, Bedford Park, South Australia 5042, Australia and* [3]*Department of Internal Medicine, South Western Medical School, University of Texas Health Science Center, Dallas, TX 75235, USA.*

**Summary** Data from a population-based case-control study conducted in Adelaide, South Australia, and involving 451 case-control pairs, were analysed to determine whether the associations of menstrual, reproductive, dietary and other factors with risk of breast cancer differed by oestrogen receptor (ER) status. Data on ER status were available for 380 cases. The proportion of tumours which were ER+ increased with age, and there was a higher proportion of ER+ tumours in post-menopausal than in premenopausal women. Both oral contraceptive use ($P=0.055$) and cigarette smoking ($P=0.047$) were associated with increased (unadjusted) risk of ER− cancer, while having little association with risk of ER+ cancer. Most dietary factors had little association with risk of either cancer type, the main exception being the reduction in risk of ER− breast cancer with increasing beta-carotene intake ($P$ for trend$=0.017$). In general, however, links with the factors examined were not strong enough to suggest different causal pathways for ER− and ER+ breast cancer.

Determination of oestrogen receptor status is used to predict the likely response of breast cancer to hormonal therapy and also has prognostic value (Hawkins *et al.*, 1980). Oestrogen receptors have been detected in 50–70% of breast cancer patients (Stanford *et al.*, 1986); two-thirds of oestrogen receptor positive (ER+) patients can be expected to respond to endocrine therapy compared to less than 10% of patients classified as oestrogen receptor negative (ER−) (Mohla *et al.*, 1982).

ER+ and ER− breast cancers differ in their biological properties. However, it has not yet been established whether ER status reflects different stages of the same disease or two different forms of the disease with separate causes (Stanford *et al.*, 1986). Complete absence of overlap between risk factors for the two types of breast cancer would provide evidence against a progression from one to the other, while overlap would suggest either that there is a progression or that they are independent outcomes with overlapping aetiologies. The few case-control studies conducted to date (Hildreth *et al.*, 1983; McTiernan *et al.*, 1986; Hislop *et al.*, 1986, 1988; Stanford *et al.*, 1987) suggest that there is some overlap in risk factors for ER+ and ER− breast cancer. This possibility, and the others outlined above, are investigated further in the population-based case-control study reported here, in which risk of ER+ and ER− breast cancer is examined in association with menstrual and reproductive history, dietary intake and other factors.

### Materials and methods

### *Study subjects*

A detailed description of the study methods has been presented elsewhere (Rohan *et al.*, 1988). Cases were women whose histologically confirmed first diagnosis of breast cancer was reported to the South Australian Central Cancer Registry between April 1982 and July 1984, who were between 20 and 74 years old at diagnosis and who were residing in the Adelaide metropolitan area and registered on the electoral roll. Of 559 eligible cases, 451 were successfully interviewed; of those not included, 15 died before interview could be arranged, 44 were deemed by the attending surgeon to be unfit for interview, 44 refused to be interviewed, and 5 were untraceable. Controls were women who had no history of breast cancer, who were also resident in the Adelaide metropolitan area and who were registered on the electoral roll. (In South Australia, 97.2% of persons eligible to vote are registered (Australian Electoral Office, 1983).) For each case, one control, matched as closely as possible to the age of the case at diagnosis, was selected at random from the electoral roll. A total of 648 individuals were approached in order to enrol 451 controls. The total study population therefore comprised 451 case-control pairs. Reasons for non-participation were recorded during the recruitment of the first 100 controls, which required attempting to recruit 151 persons. Of the 51 who did not participate, 39 refused, 11 were untraceable and one had died.

### *Data collection procedure*

Subjects were interviewed in their own homes by trained interviewers whose performance was monitored regularly. Interviewers were assigned randomly to case-control pairs. For cases, the average interval between diagnosis and interview was 4.8 months. Each control was interviewed as soon as possible after her case had been enrolled and in a few instances interview of the control preceded that of her case. Approximately 90% of controls were interviewed within two months of the corresponding case. Matching on date of interview was introduced in an attempt to minimise dietary differences between cases and controls due to seasonal influences.

Socio-demographic and medical information was collected by use of an interviewer-administered questionnaire which sought basic biographic information, personal medical history, family history of cancer, gynaecological and reproductive history and history of hormone use.

Information on usual dietary intake was collected from the study participants by means of a self-administered quantitative food frequency questionnaire. The questionnaire, which was designed to ascertain total daily intake of energy, several nutrients, alcohol and methylxanthines, has been described in detail elsewhere (Baghurst & Baghurst, 1981; Baghurst and Record, 1983). Briefly, the questionnaire allowed recording of the usual frequency of consumption of 179 specified dietary items; provided space for participants to indicate whether their usual serving size for an item differed from that of a specified standard serving size (Baghurst & Record, 1983; Thomas & Corden, 1970), and also provided space for them to indicate consumption of other items not listed in the main body of the questionnaire. Additional data recorded and used in the derivation of

Correspondence: J.A. Cooper.
Received 31 May 1988; and in revised form, 30 August 1988.

estimates of nutrient and energy intake (see below) included information on cooking practices; use of sugar, salt and fat; and bread type.

Data on the frequency of consumption of food items were converted to daily nutrient, alcohol, methylxanthine and energy intake by a computerised dietary analysis system (Baghurst & Baghurst, 1981; Baghurst & Record, 1984). The food frequency questionnaire has been shown to provide estimates of nutrient and energy intake which are repeatable (Rohan *et al.*, 1987), and which are similar to those derived from studies in Australia in which dietary records have been used (Baghurst & Baghurst, 1981). A similar food frequency questionnaire has been shown to have repeatability and criterion validity (Willett *et al.*, 1985).

*Preparation of tumour cytosol fractions*

Frozen tumour tissue was powdered by percussion and then homogenised (ultraturrax) in ice-cold buffer containing 10 M Tris, 1.5 M EDTA, 1 mM dithiothreitol, 10% glycerol and 20 mM sodium molybdate, pH 7.4. The final cytosol fractions were obtained by centrifugation of the homogenates at 105,000*g* for 1 h at 4°C.

*Hormone receptor determination*

Receptor levels for oestrogen and progesterone (results not shown – see below) were measured using saturation analysis assays. Five incubation concentrations, ranging from 0.05 to 2.0 nM for $^3$H-oestradiol and from 0.08 to 8.0 nM for $^3$H-R5020, were used to determine the total ER and progesterone receptor (PR) binding, respectively, to the tumour cytosol fraction. Parallel series of incubations containing the radioligands in the presence of a 100-fold excess of appropriate unlabelled ligand (diethylstilboestrol for $^3$H-oestradiol and R5020 for $^3$H-R5020) were used to estimate the levels of non-specific binding. Incubations were conducted in duplicate, in microtitre plates, with a final incubation volume of 100 $\mu$l. Following a 16 h incubation at 4°C, bound and free hormone were separated by the addition of dextran-coated charcoal. Binding data were analysed according to the method of Scatchard, with least squares linear regression analysis (Horsfall *et al.*, 1986).

Receptor concentrations were expressed as fmol mg$^{-1}$ cytosol protein. Tumour cytosols with a receptor concentration equal to or greater than 10 fmol mg$^{-1}$ protein were graded as positive in this study. This cut-off was applied to both ER and PR concentrations. All of the assays were performed in one laboratory.

*Statistical analysis*

ER status was determined for 380 cases, PR for 377. No difference with respect to socio-demographic characteristics and the examined risk factors was found between those for whom ER status was known and those for whom it was unknown (for example – years of education ($\chi^2_2=0.138$, $P=0.933$), age ($\chi^2_4=6.82$, $P=0.146$), menopausal status ($\chi^2_1=0.69$, $P=0.406$), family history ($\chi^2_1=0.042$, $P=0.838$) and oral contraceptive (OC) use ($\chi^2_1=0.07$, $P=0.791$).

Two types of analyses were used to study the association between risk factors and ER status. In one, cases only were employed in logistic regression models in which ER status (ER+ versus ER−) was used as the response variable. This strategy was used to examine the associations of age and menopausal status with risk of ER+ versus ER− breast cancer. (The criteria used to define menopausal status have been described elsewhere (Rohan *et al.*, 1988).) It was also used to determine whether the associations between the factors of interest and risk of breast cancer differed by ER status; for this purpose, the change in deviance associated with the addition of the factor to the model formed a $\chi^2$ test on $n-1$ degrees of freedom, where $n$ is the number of levels at which the factor was examined. The remaining analysis involved case-control comparisons conducted within strata defined by ER status. For these analyses, conditional logistic regression models (Breslow & Day, 1980) were used to derive relative risks (RR) and 95% confidence intervals (CI) for the association between the factors of interest and (ER+ or ER−) breast cancer. The univariate association of each factor was examined before the effects of potential confounders were assessed in multiple conditional logistic regression models. Interactions with menopausal status were also examined, and the only interaction which was statistically significant was that for cigarette smoking. All tests of statistical significance were two-sided.

Factors studied included menstrual and reproductive factors, family history of breast cancer in a first-degree relative, previous benign breast disease, hormone use, cigarette smoking and several dietary factors. Analyses for nutrients were based initially on unadjusted estimates of intake and were then repeated after adjusting these estimates for caloric intake by the method of Willett *et al.* (1985). For these analyses the dietary factors were categorised by tertiles derived from their distribution in the controls.

ER concentration was positively associated with age (see below), and therefore analyses were repeated using age-adjusted values for ER concentration. The results of these analyses differed little from those based on the unadjusted values, and only the latter are presented here. Also, ER and PR status were strongly correlated (62% of ER+ tumours were PR+, and 78% of ER− tumours were PR−). Therefore, results based on PR status were similar to those based on ER status, and only the latter are presented.

### Results

*Age and menopausal status*

Of the 380 cases for whom ER status was determined, 251 were ER+ and 129 were ER−. Table I, which displays the distribution of cases by ER status, age and menopausal status, shows that the proportion of breast cancers which were ER+ increased with age. A logistic regression model fitted to case data only showed tumours to be 4.52 times more likely to be ER+ in the oldest age group than in the youngest (95% CI 2.26–9.09). Table I also shows that there was also a strong relationship between ER status and menopausal status, with 74.0% of post-menopausal cancers being ER+ compared to 52.1% of premenopausal cancers. In further logistic modelling using the case data only, ER status had a statistically significant association with menopausal status when the latter was fitted independently; ER+ breast cancer was 2.61 times more common in postmenopausal than in premenopausal women (RR 2.61, 95% CI 1.65–4.14). However, neither age nor menopausal status added significantly to the model once the other had been fitted. These patterns were also observed in analyses in which the actual ER concentration of the tumour was used (with $\log_{10}$ ER concentration as the response variable).

In an attempt to separate the effects of age and menopausal status, the effect of menopausal status was studied in women aged 45–54 years, the only age group containing both premenopausal (47 cases) and post-menopausal (32 cases) women. In this age group, menopausal status was not associated with altered risk of either ER+ or ER− breast cancer.

*Menstrual and reproductive factors*

Risk of ER+ breast cancer increased (albeit not significantly) with age at first full-term pregnancy, risk for nulliparous women being slightly less than that for women with a first full-term pregnancy at 30 years of age or later (Table II). For ER− breast cancer, nulliparous women and women with a first full-term pregnancy at age 20 or later were all at increased risk (relative to the risk for women with an early first full-term pregnancy), but there was no evidence of a linear relationship between age at first full-term pregnancy

**Table I** Distribution of breast cancer cases by ER status, age and menopausal status

| | *Premenopausal cases* | | *Post-menopausal cases* | | *Total* | |
|---|---|---|---|---|---|---|
| *Age* | *ER+* | *ER−* | *ER+* | *ER−* | *ER+* | *ER−* |
| 20–44 | 34 (47.2%) | 38 (52.8%) | 2 (100%) | 0 | 36 (48.7%) | 38 (51.3%) |
| 45–54 | 28 (59.6%) | 19 (40.4%) | 22 (68.8%) | 10 (31.2%) | 50 (63.3%) | 29 (36.7%) |
| 55–64 | 0 | 0 | 81 (69.8%) | 35 (30.2%) | 81 (69.8%) | 35 (30.2%) |
| 65–74 | 0 | 0 | 71 (80.7%) | 17 (19.3%) | 71 (80.7%) | 17 (19.3%) |
| Total | 62 (52.1%) | 57 (47.9%) | 176 (74%) | 62 (26.1%) | 238 (66.7%) | 119 (33.3%) |

Of the 380 cases for whom ER status was determined, 22 (12 ER+ and 10 ER−) were deemed to be perimenopausal, while for the remaining individual menopausal status could not be determined; per cent distribution is shown in parentheses.

**Table II** Relative risk of ER+ and ER− breast cancer by menstrual and reproductive factors

| | | *ER+* | | | *ER−* | | | *P value for test of difference between ER groups* |
|---|---|---|---|---|---|---|---|---|
| *Factor* | *Level* | *No. of cases* | *No. of controls* | *RR (95% CI)* | *No. of cases* | *No. of controls* | *RR (95% CI)* | |
| Age (years) | <20 | 30 | 27 | 1.0[a] | 10 | 13 | 1.0[a] | |
| at first | 20–24 | 69 | 81 | 1.07 (0.54–2.11) | 52 | 43 | 2.42 (0.99–5.90) | |
| full-term | 25–29 | 80 | 68 | 1.26 (0.64–2.49) | 43 | 40 | 1.15 (0.47–2.81) | 0.393 |
| pregnancy | >29 | 66 | 69 | 1.55 (0.69–3.48) | 24 | 33 | 1.25 (0.39–4.01) | |
| | Nulliparous | 5 | 5 | 1.44 (0.67–3.11) | 1 | 1 | 1.67 (0.55–5.06) | |
| | *P* value for linear trend | | | 0.206 | | | 0.731 | |
| Parity | Nulliparous | 39 | 35 | 1.0[a] | 15 | 15 | 1.0[a] | |
| | 1 | 43 | 27 | 1.42 (0.74–2.72) | 20 | 15 | 1.25 (0.45–3.43) | |
| | 2 | 61 | 70 | 0.80 (0.46–1.37) | 38 | 47 | 0.81 (0.35–1.91) | 0.403 |
| | >2 | 107 | 118 | 0.82 (0.48–1.40) | 57 | 53 | 0.99 (0.44–2.25) | |
| | *P* value for linear trend | | | 0.146 | | | 0.844 | |
| Total months | 0 | 73 | 67 | 1.0[a,b,c] | 45 | 39 | 1.0[a,b,c] | |
| lactated[b] | 1–12 | 108 | 114 | 0.97[b] (0.54–1.72) | 60 | 61 | 0.73[b] (0.37–1.45) | 0.300 |
| | >12 | 69 | 69 | 1.05[b] (0.59–2.10) | 25 | 30 | 0.64[b] (0.29–1.43) | |
| | *P* value for linear trend | | | 0.83 | | | 0.28 | |
| Age (years) | <13 | 90 | 90 | 1.0[a] | 52 | 43 | 1.0[a] | |
| at menarche | 13 | 60 | 63 | 0.93 (0.58–1.50) | 36 | 37 | 0.86 (0.60–1.65) | 0.951 |
| | >13 | 97 | 94 | 1.03 (0.59–2.10) | 42 | 50 | 0.76 (0.40–1.44) | |
| | *P* value for linear trend | | | 0.965 | | | 0.403 | |
| Age (years) | <47 | 57 | 71 | 1.0[a,d] | 15 | 23 | 1.0[a,d] | |
| at last | 47–50 | 49 | 51 | 1.28 (0.76–2.16) | 24 | 22 | 2.35 (0.87–6.38) | 0.259 |
| menstrual | >50 | 65 | 51 | 1.85 (1.04–3.29) | 23 | 25 | 1.96 (0.73–5.25) | |
| period | *P* value for linear trend | | | 0.034 | | | 0.236 | |

[a]Reference category. RR estimates for age at first full-term pregnancy, parity total months lactated and age at menarche are adjusted for menopausal status. *Note:* RR are matched and cannot be calculated directly from the unmatched distributions of cases and controls shown in the table; [b]Total months lactated is adjusted for menopausal status, parity, age at first birth; [c]Reference category includes nulliparous women; [d]Restricted to post-menopausal women.

and risk of breast cancer. A parity of two or more was associated with a small, statistically non-significant reduction in risk of ER+ breast cancer, while for ER− breast cancer only women who had had two full-term pregnancies were at reduced risk of breast cancer. There was relatively little variation in the risk of either ER+ or ER− breast cancer with age at menarche and total duration of lactation, although there was some suggestion that risk of ER− breast cancer decreased with increasing duration of lactation and with age at menarche. Estimates of effect for duration of lactation were adjusted for parity and age at first birth (Table II).

Post-menopausal women were at lower risk of ER+ and ER− breast cancer than premenopausal women. The risk of ER+ cancer for post-menopausal women relative to that for premenopausal women was 0.86 (95% CI 0.29–2.55). The corresponding relative risk for ER− cancer was 0.29 (0.06–1.38). A relatively late age at last menstrual period was associated with increased risk of both ER+ and ER− breast cancer, and for the former group there was a statistically significant trend of increasing risk with increasing age at last menstrual period.

### *History of benign breast disease and family history of breast cancer*

Women with a history of benign breast disease were at increased risk of ER+ breast cancer and at decreased risk of ER− breast cancer (Table III). However, the confidence intervals associated with both estimates of effect were wide. Also, a family history of breast cancer was associated with an increased, but statistically non-significant, risk of both ER+ and ER− tumours (Table III).

### *Hormone use*

Ever use of oral contraceptives (OCs) was not associated with altered (unadjusted) risk of ER+ breast cancer (RR 0.91, 95% CI 0.56–1.78), but was associated with a (statistically non-significant) 70% increase in (unadjusted) risk of ER− breast cancer (RR 1.68, 95% CI 0.84–3.35). The difference between these associations was statistically significant ($P = 0.028$). After adjustment for age at first birth, parity and smoking this difference remained, but the estimate of effect for ER− breast cancer was markedly reduced (Table III). Ever use of replacement oestrogens was not associated with statistically significant alterations in the risk of either cancer type (Table III), although risk of ER− breast cancer was increased by 80%. For neither type of exogenous hormone was there a clear pattern of variation in risk with duration of exposure (results not shown).

### *Cigarette smoking*

Women who had ever smoked cigarettes had an increased risk of ER− breast cancer (Table III), a marginally statis-

**Table III** Relative risk of breast cancer by selected risk factors

| Factor | Level | ER+ No. of cases | ER+ No. of controls | ER+ RR (95% CI) | ER− No. of cases | ER− No. of controls | ER− RR (95% CI) | P value for test of difference between ER groups |
|---|---|---|---|---|---|---|---|---|
| History of benign breast disease | No | 235 | 243 | 1.0[a] | 124 | 122 | 1.0[a] | 0.439 |
| | Yes | 14 | 6 | 2.56 (0.90–7.29) | 5 | 8 | 0.44 (0.09–2.23) | |
| History of breast cancer in first degree relative | No | 226 | 234 | 1.0[a] | 119 | 124 | 1.0[a] | 0.752 |
| | Yes | 24 | 16 | 1.60 (0.83–3.09) | 11 | 6 | 1.49 (0.52–4.26) | |
| Ever use of oral contraceptives (OCs) | Never | 161 | 158 | 1.0[a,b] | 54 | 63 | 1.0[a,b] | 0.01 |
| | Ever | 88 | 92 | 0.88 (0.53–1.46) | 76 | 67 | 1.33 (0.69–2.55) | |
| Ever use of replacement oestrogens | Never | 205 | 195 | 1.0[a] | 105 | 101 | 1.0[a] | 0.584 |
| | Ever | 41 | 50 | 0.85 (0.53–1.38) | 24 | 26 | 1.84 (0.46–2.20) | |
| Ever smoked cigarettes | Never | 149 | 150 | 1.0[a] | 66 | 82 | 1.0[a] | 0.036 |
| | Ever | 101 | 100 | 0.95 (0.66–1.37) | 64 | 48 | 1.63 (1.00–2.66) | |
| Current smoking status | Never | 149 | 150 | 1.0[a] | 66 | 82 | 1.0[a] | 0.150 |
| | Current | 54 | 46 | 1.25 (0.78–1.99) | 32 | 28 | 1.33 (0.71–2.51) | |
| | Ex | 47 | 54 | 0.88 (0.56–1.38) | 32 | 20 | 1.89 (0.99–3.64) | |

[a]Reference category. All RR estimates adjusted for menopausal status. *Note:* RR are matched and cannot be calculated directly from the unmatched distributions of cases and controls shown; [b]Estimates for OC use also adjusted for parity, age at first birth and cigarette smoking.

tically significant association which remained after controlling for obesity, hormone use and reproductive factors, and which differed significantly ($P=0.036$) from the association between cigarette smoking and risk of ER+ breast cancer. The increase in risk was confined largely to ex-smokers (defined as women who had last smoked more than one year ago). For ER− breast cancer there was a statistically significant interaction between smoking and menopausal status ($\chi^2_2=3.93$, $P=0.047$), the effect of smoking being greater among premenopausal women. The risk for ever smokers relative to that for never smokers was 3.05 (1.29–7.18) for premenopausal women and 1.04 (0.18–5.96) for post-menopausal women.

### *Obesity and dietary factors*

Relative risks for ER+ and ER− breast cancer by obesity and by selected dietary factors are shown in Table IV. Neither obesity, nor intake of protein, fat, alcohol or methylxanthines was associated with marked alterations in risk of either cancer type. Risk was slightly increased at the second level of energy intake for ER+ cancer, and decreased at the corresponding level for ER− cancer, but these point estimates were not statistically significant. The main differences between tumour types were the findings for beta-carotene and retinol intake, both having stronger associations with risk of ER− cancer, and relatively little association with risk of ER+ breast cancer. A relatively high retinol intake was associated with a statistically non-significant increased risk of ER− cancer, risk for the mid-level of intake differing little from baseline risk. Risk of ER− breast cancer decreased with increasing beta-carotene intake, a trend which was statistically significant ($P=0.017$).

The patterns described above were mostly similar when the analyses were repeated using nutrient intake adjusted for total caloric intake. The main differences were for retinol and beta-carotene intake. For retinol, risk of ER− breast cancer now increased with increasing intake, risks for medium and high levels of intake being, respectively, 1.21 (95% CI 0.65–2.25) and 1.70 (0.87–3.33); however, the trend in risk was not statistically significant ($P=0.107$). In contrast, there were now statistically significant trends of decreasing risk of ER+ and ER− breast cancer with increasing beta-carotene intake; for ER− breast cancer, relative risks at medium and high levels of intake were 0.69 (0.38–1.27) and 0.33 (0.16–0.68), respectively ($P$ value for linear trend, 0.002), while for ER+ breast cancer, corresponding relative risks were 0.78 (0.50–1.21) and 0.60 (0.38–0.94) ($P$ value for linear trend, 0.024).

### Discussion

Possible sources of bias in this study have been discussed in detail elsewhere (Rohan *et al.*, 1988). In brief, selection bias may have arisen from non-response by potential cases and controls. However, included and non-included cases did not differ in their distributions by socio-demographic (age, socio-economic grading of area of residence) and tumour characteristics (diameter, number of nodes). Data on the ER status of the non-included cases are not available, but given the similar age distributions of the included and non-included cases, there is no firm evidence that cases were selected by ER status. Further, although ER data were not available for all included cases, there were no differences between those for whom ER status was known and those for whom it was unknown with respect to socio-demographic characteristics and the risk factors examined in this study. In the controls, response rates decreased with age (from 88% in those aged 20–34 years to 61% in those aged 65–74 years). The consequences of this cannot be predicted, and the possibility of selection bias cannot be excluded. Misclassification of subjects with respect to exposure is another important potential source of bias here. Among cases, there is no *a priori* reason to suspect that the likelihood and magnitude of this differed by ER status.

In this study, age and menopausal status had strong associations with ER status, ER+ breast cancer being more common in older than in younger women and more common in post-menopausal than in premenopausal women. The same patterns have been observed in many previous studies (Stanford *et al.*, 1986). Although analyses in the subgroup of women aged 45–54 years in the present study suggested that age is the more important of these two confounded factors, a finding in agreement with the results of several other studies (e.g. Elwood & Godolphin, 1980; Hulka *et al.*, 1984; McCarty *et al.*, 1983), separation of the effects of these two highly correlated variables is difficult (Stanford *et al.*, 1986). The reason why the proportion of tumours which are ER+ is higher in older (post-menopausal) women than in younger (premenopausal) women is unknown, but may be due to the lower plasma levels of endogenous oestrogens in the former group, with consequent relative underoccupation of ER sites, and also to the lower plasma levels of progesterone in this

**Table IV** Relative risk of ER+ and ER− breast cancer by obesity and by absolute daily intake of selected dietary factors

| Factor | Level | ER+ No. of cases | ER+ No. of controls | ER+ RR (95% CI) | ER− No. of cases | ER− No. of controls | ER− RR (95% CI) | P value for test of difference between ER groups |
|---|---|---|---|---|---|---|---|---|
| Quetelet's | <22.8 | 85 | 89 | 1.0[a] | 43 | 40 | 1.0[a] | |
| index (kg m$^{-2}$) | 22.8–26.0 | 69 | 76 | 0.99 (0.64–1.52) | 38 | 46 | 0.68 (0.36–1.28) | 0.472 |
| | >26.0 | 94 | 83 | 1.24 (0.79–1.94) | 49 | 42 | 0.99 (0.55–1.80) | |
| | P value for linear trend | | | 0.36 | | | 0.94 | |
| Energy | <6906.7 | 77 | 82 | 1.0[a] | 48 | 43 | 1.0[a] | |
| (kJ day$^{-1}$) | 6906.7–8877.0 | 92 | 84 | 1.20 (0.76–1.88) | 33 | 42 | 0.73 (0.39–1.36) | 0.165 |
| | >8877.0 | 81 | 84 | 1.05 (0.80–1.68) | 49 | 45 | 1.10 (0.61–2.00) | |
| | P value for linear trend | | | 0.904 | | | 0.813 | |
| Protein | <68.7 | 76 | 90 | 1.0[a] | 44 | 38 | 1.0[a] | |
| (g day$^{-1}$) | 68.7–89.0 | 84 | 77 | 1.25 (0.80–1.89) | 34 | 46 | 0.69 (0.37–1.28) | 0.387 |
| | >89.0 | 90 | 83 | 1.31 (0.85–2.03) | 52 | 46 | 1.10 (0.59–2.04) | |
| | P value for linear trend | | | 0.225 | | | 0.773 | |
| Total fat | <66.5 | 79 | 84 | 1.0[a] | 40 | 39 | 1.0[a] | |
| (g day$^{-1}$) | 66.5–92.7 | 89 | 78 | 1.21 (0.76–1.91) | 47 | 49 | 1.04 (0.57–1.92) | 0.999 |
| | >92.7 | 82 | 88 | 1.31 (0.85–2.03) | 43 | 42 | 1.15 (0.60–2.19) | |
| | P value for linear trend | | | 0.958 | | | 0.792 | |
| Alcohol | Non-drinker | 93 | 96 | 1.0[a] | 42 | 43 | 1.0[a] | |
| (g day$^{-1}$) | <2.5 | 44 | 45 | 1.02 (0.58–1.77) | 22 | 33 | 0.56 (0.28–1.12) | 0.777 |
| | 2.5–9.3 | 45 | 53 | 0.89 (0.53–1.89) | 27 | 25 | 1.06 (0.52–2.21) | |
| | >9.3 | 68 | 56 | 1.28 (0.77–2.13) | 39 | 29 | 1.21 (0.58–2.51) | |
| | P value for linear trend | | | 0.440 | | | 0.491 | |
| Retinol | <334.4 | 90 | 81 | 1.0[a] | 40 | 45 | 1.0[a] | |
| (μg day$^{-1}$) | 334.4–962.8 | 81 | 83 | 0.89 (0.56–1.42) | 30 | 41 | 0.85 (0.46–1.56) | 0.014 |
| | >962.8 | 79 | 86 | 0.78 (0.49–1.25) | 60 | 44 | 1.66 (0.91–3.01) | |
| | P value for linear trend | | | 0.394 | | | 0.119 | |
| Beta-carotene | <4152.7 | 88 | 82 | 1.0[a] | 55 | 43 | 1.0[a] | |
| (μg day$^{-1}$) | 4152.7–7196.8 | 93 | 77 | 1.09 (0.71–1.68) | 47 | 41 | 0.81 (0.44–1.51) | 0.427 |
| | >7196.8 | 69 | 91 | 0.71 (0.46–1.11) | 28 | 46 | 0.43 (0.21–0.88) | |
| | P value for linear trend | | | 0.182 | | | 0.017 | |
| Methyl- | <233.6 | 86 | 84 | 1.0[a] | 43 | 43 | 1.0[a] | |
| xanthines | 233.6–349.2 | 72 | 83 | 0.95 (0.49–2.04) | 40 | 43 | 0.95 (0.49–2.04) | 0.861 |
| (mg day$^{-1}$) | >349.2 | 92 | 83 | 1.26 (0.68–2.31) | 47 | 44 | 1.26 (0.68–2.31) | |
| | P value for linear trend | | | 0.758 | | | 0.559 | |

[a]Reference category. All RR estimates adjusted for menopausal status. *Note:* RR are matched and cannot be calculated directly from the unmatched distributions of cases and controls shown in the table.

group, with consequent reduction in the inhibitory effect of progesterone on ER synthesis (Hawkins *et al.*, 1980).

Many of the other factors investigated here were confounded by menopausal status and age, and had relatively weak associations with risk of ER+ and ER− breast cancer. In this respect, the results presented here are largely in accord with those of previous case-control studies which have examined risk factors for breast cancer by ER status (Hildreth *et al.*, 1983; McTiernan *et al.*, 1986; Hislop *et al.*, 1986, 1988; Stanford *et al.*, 1987), and the general absence of statistically significant associations may reflect the relatively low statistical power of studies to date.

Patterns of risk in association with the variables examined in the present study were mostly similar for ER+ and ER− breast cancer (and, in general, this applies also to the results of previous case-control studies), exceptions being the findings for a history of benign breast disease, for ever use of oral contraceptives, for cigarette smoking and for intake of retinol and beta-carotene. The wide confidence intervals around the point estimates for risk in association with a history of benign breast disease suggest that, for this variable at least, chance may provide an explanation for the divergent results. Nevertheless, results in accord with those observed here (i.e. increased risk of ER+ breast cancer and decreased risk of ER− breast cancer) were also reported by Hildreth *et al.* (1983).

In the present study, ever use of OCs was associated with a statistically non-significant increase in the risk of ER− breast cancer, and with little alteration in the risk of ER+ breast cancer. Results of previous studies are summarised in Table V. Neither of the previous case-control studies showed pronounced effects of OCs, while of the four studies involving breast cancer cases only (Elwood & Godolphin, 1980; Hulka *et al.*, 1984; Lesser *et al.*, 1981; Osborne *et al.*, 1983), two (Lesser *et al.*, 1981; Osborne *et al.*, 1983) showed those who had ever used OCs to be more likely to have ER− tumours, while the remaining studies showed no difference between ever and never users of OCs. Collectively, therefore, studies to date do not provide strong support for an association between OC use and ER status. It is possible that this reflects a counterbalancing effect of progesterone on the effects of oestrogen (Stanford *et al.*, 1986). The present study also showed a statistically non-significant positive association between replacement oestrogens and risk of ER− breast cancer. This observation provides qualified support for the hypothesis that users of exogenous oestrogens are likely to develop ER− breast cancer due to occupation of binding sites by the exogeneous oestrogen. Replacement oestrogens and OCs might be expected to differ in their effects, not only because of absence of progesterone in the former, but also because synthetic oestrogens (which are usually present in OCs) are more potent than natural oestrogens (which are usually used for replacement purposes) (Longman & Buehring, 1987).

Women who had ever smoked cigarettes had increased risk of ER− breast cancer, but analtered risk of ER+ breast cancer. These findings are consistent with an 'anti-oestrogenic' effect of cigarette smoking, as proposed by Baron (1984). A possible explanation for this is competitive binding to oestrogen receptors by a constituent of cigarette smoke (Baron, 1984). However, the association between smoking and risk of ER− breast cancer was stronger for ex-

**Table V** Oral contraceptives, oestrogen receptor status and breast cancer: a summary

| Reference | Type of analysis | No. of subjects ER+ | No. of subjects ER− | Confounding variables considered | Contrast | Results | |
|---|---|---|---|---|---|---|---|
| | | | | | | RR (95% CI) | |
| | | | | | | ER+ | ER− |
| Stanford *et al.* (1987) | Case-control | 204 | 254 | race, menopausal status, family history, benign breast disease, quetelet's index | Ever *vs.* never used | 0.84 (0.58–1.22) | 1.22 (0.84–7.75) |
| | | | | | | RR (95% CI) | |
| | | | | | | ER+ | ER− |
| McTiernan *et al.* (1986) | Case-control | 143 | 97 | age | Ever *vs.* never used | 1.2 (0.74–1.9) | 0.82 (0.47–1.4) |
| | | | | | | Odds of ER+ tumour | |
| Elwood & Godolphin (1980) | Case only | 526 | 145 | age | Ever *vs.* never used | 0.83 | $P=0.54$ |
| | | | | | | Median receptor level ($fmol\,mg^{-1}$) | |
| Hulka *et al.* (1984) | Case only | 246 | | race, age | Never used<br>Ever used | 19.1<br>18.5 | $P=0.65$ |
| | | | | | | Median receptor level ($fmol\,mg^{-1}$) | |
| Lesser *et al.* (1981) | Case only | 397 | 284 | – | Never used<br>Ever used | 11<br>6 | $P<0.001$ |
| | | | | | | % ER− | |
| Osborne *et al.* (1983) | Case only | 47 | 71 | family history | Never used<br>Ever used | 55%<br>70% | $P=0.04$ |

smokers, which raises the possibility that hormonal changes associated with smoking cessation may permanently change the hormone binding property of malignant tissue. An alternative explanation for these findings is confounding by an unmeasured variable.

Obese women are at increased risk of developing breast cancer (Kelsey, 1979). Additionally, heavy women with breast cancer have been observed to have poor prognoses (Donegan *et al.*, 1978; Boyd *et al.*, 1981). It is not known how obesity exerts these effects, but an influence on endocrine status offers a plausible explanation. Obesity is associated with increased peripheral production of oestrone in adipose (and other) tissue, and is also associated with reduced levels of sex hormone-binding globulin, so that obese women have increased levels of free (bioactive) oestrogens (Henderson *et al.*, 1982). Given these endocrine effects of obesity, and given that oestrogen influences the development of its own receptor (Stanford *et al.*, 1986), a relationship between obesity and the ER status of breast tumours might be anticipated. From a clinical study of 83 women with breast cancer, Papatestas *et al.* (1980) reported that tumours from women of relatively high body weight (>150 lb) had lower ER levels than those from women of lower weight (<150 lb). They suggested that this observation might explain the less favourable clinical characteristics of breast cancer in the obese, and that it might reflect an underlying role for dietary factors. Results from case-control studies of obesity, ER status and risk of breast cancer have not been consistent, however. In the present study, obesity was associated with very little alteration in the risk of ER+ or ER− breast cancer. Previous studies have shown that either positive associations between obesity and risk of ER+ and ER− breast cancer (Stanford *et al.*, 1987), or an inverse association between weight and risk of ER− breast cancer, with no association between weight and risk of ER+ breast cancer (Hislop *et al.*, 1986).

The relationship between dietary factors and risk of breast cancer by oestrogen receptor status has been examined in only one previous case-control study. Hislop *et al.* (1988) found that risk of ER+ and ER− breast cancer increased with increasing frequency of consumption of various sources of meat fat, suggesting that if there is a relationship between dietary fat and risk of breast cancer, it is not mediated by ER status. There were no clear trends in risk with consumption of green and yellow vegetables. In contrast, in the present study, while there was no association between energy, protein or fat intake and risk of ER+ or ER− breast cancer, some support for an association between diet and ER status was provided by the results for vitamin A. Both retinol and beta-carotene were found to have stronger associations with ER− cancer. Specifically, while retinol and beta-carotene had weak associations with risk of ER+ breast cancer, there was a statistically significant reduction in risk of ER− breast cancer with increasing intake of beta-carotene, and some suggestion of an increase in risk of ER− breast cancer at high levels of retinol intake. The latter finding is contrary to the experimental evidence relating retinol to reduced risk of cancer (Wald, 1987), and may represent a chance effect. In contrast, our finding of reduced risk of ER− breast cancer at high levels of beta-carotene intake is in accord with the results of the many dietary and serum studies which have shown an inverse association between beta-carotene and cancer risk (Wald, 1987). There is, however, little evidence relating dietary beta-carotene to risk of breast cancer (Rohan *et al.*, 1988). Study of beta-carotene is in effect a study of green and yellow vegetable intake, and it is uncertain whether findings for beta-carotene reflect a direct effect or an indirect effect of some other factor associated with beta-carotene (Wald, 1987). Absence of an association between dietary fat and ER status in the two case-control studies to date runs counter to experimental evidence suggesting higher levels of ER in association with a relatively high fat intake (Ip & Ip, 1981). Given the suspected role of dietary factors in breast cancer aetiology (Rohan & Bain, 1987) and in influencing survival from breast cancer (via an effect on obesity), and given that at least some dietary factors (and, in particular, dietary fat) might operate via an influence on ER status (Rohan & Bain,

1987), further research into the links between diet and ER status is warranted, since it might yield preventive and therapeutic opportunities.

In conclusion, this study has not provided evidence consistent with there being different causal pathways for ER+ and ER− cancer. Indeed, from the results of studies to date, this seems unlikely, since both tumour types seem to share risk factors, although some of the risk factors have stronger associations with one or the other of the two tumour subtypes (Stanford *et al.*, 1986). These observations are consistent with the prediction of Moolgavkar *et al.* (1980) that malignant breast tumours are initially ER+, but subsequently become hormone independent through clonal evolution. However, the possibility that ER+ and ER− breast cancers represent independent outcomes with overlapping aetiologies cannot be excluded.

These data were collected while Dr Rohan was with the Commonwealth Scientific and Industrial Research Organization Division of Human Nutrition, Adelaide, Australia. The authors thank the referees, and Drs John Potter and John Whitehead for their helpful comments on the manuscript.

### References

AUSTRALIAN ELECTORAL OFFICE (1983). A quantitative assessment of electoral enrolment in Australia. Research Report, Australian Electoral Office: Canberra.

BAGHURST, K.I. & BAGHURST, P.A. (1981). The measurement of usual dietary intake in individuals and groups. *Trans. Menzies Found.*, **3**, 139.

BAGHURST, K.I. & RECORD, S.J. (1983). Intakes and sources, in selected Australian populations, of dietary constituents implicated in the etiology of chronic diseases. *J. Food Nutr.*, **40**, 1.

BAGHURST, K.I. & RECORD, S.J. (1984). A computerised dietary analysis system for use with diet diaries of food frequency questionnaires. *Commun. Health Stud.*, **8**, 11.

BARON, J.A. (1984). Smoking and estrogen-related disease. *Am. J. Epidemiol.*, **119**, 9.

BOYD, N.F., CAMPBELL, J.E., GERMANSON, T., THOMSON, D.B., SUTHERLAND, D.J. & MEAKIN, J.W. (1981). Body weight and prognosis in breast cancer. *J. Natl Cancer Inst.*, **67**, 785.

BRESLOW, N.E. & DAY, N.E. (1980). *Statistical Methods in Cancer Research, Vol. 1, The Analyses of Case-control Studies.* IARC Scientific Publications: Lyon.

DONEGAN, W.L., HARTZ, A.J. & RIMM, A.A. (1978). The association of body weight with recurrent cancer of the breast. *Cancer*, **41**, 1590.

ELWOOD, J.M. & GODOLPHIN, W. (1980). Oestrogen receptors in breast tumours: Associations with age, menopausal status and epidemiological and clinical features in 735 patients. *Br. J. Cancer*, **42**, 635.

HAWKINS, R.A., ROBERTS, M.M. & FORREST, A.P.M. (1980). Oestrogen receptors and breast cancer: Current status. *Br. J. Surg.*, **67**, 153.

HENDERSON, B.E., ROSS, R.K., PIKE, M.C. & CASAGRANDE, J.T. (1982). Endogenous hormones as a major factor in human cancer. *Cancer Res.*, **42**, 3232.

HILDRETH, N.G., KELSEY, J.L., EISENFELD, A.J., LI VOLSI, V.A., HOLFORD, T.R. & FISCHER, D.B. (1983). Differences in breast cancer risk factors according to the estrogen receptor level of the tumour. *J. Natl Cancer Inst.*, **70**, 1027.

HISLOP, T.G., COLDMAN, A.J., ELWOOD, J.M., SKIPPEN, D.H. & KAN, L. (1986). Relationship between risk factors for breast cancer and hormonal status. *Int. J. Epidemiol.*, **15**, 469.

HISLOP, T.G., KAN, L., COLDMAN, A.J., BAND, P.R. & BRAUER, G. (1988). Influence of estrogen receptor status on dietary risk factors for breast cancer. *Can. Med. Assoc. J.*, **138**, 424.

HORSFALL, D.J., TILLEY, W.D., ORELL, S.R., MARSHALL, V.R. & CANT, E.L. McK. (1986). Relationship between ploidy and steroid hormone receptors in primary invasive breast cancer. *Br. J. Cancer*, **53**, 23.

HULKA, B.S., CHAMBLESS, L.E., WILKINSON, W.E., DELIBNER, D.C., McCARTY, K.S. JNR. & McCARTY, K.S. SNR. (1984). Hormonal and personal effects on estrogen receptors in breast cancer. *Am. J. Epidemiol.*, **119**, 692.

IP, C. & IP, M.M. (1981). Serum estrogens and estrogen responsiveness in 7,12-dimethylbenz [a] anthracene-induced mammary tumors as influenced by dietary fat. *J. Natl Cancer Inst.*, **66**, 291.

KELSEY, J.L. (1979). A review of the epidemiology of human breast cancer. *Epidemiol. Rev.*, **1**, 74.

LESSER, M.L., ROSEN, P.P., SENIE, R.T., DUTHIE, K., MENENDEZ-BOTET, C. & SCHWARTZ, M.K. (1981). Estrogen and progesterone receptors in breast carcinoma: Correlations with epidemiology and pathology. *Cancer*, **48**, 299.

LONGMAN, S.M. & BUEHRING, G.C. (1987). Oral contraceptives and breast cancer. *In vitro* effect of contraceptive steroids on human mammary cell growth. *Cancer*, **59**, 281.

McCARTY, K.S. JNR., SILVA, J.S., COX, E.B., LEIGHT, G.S. JNR., WELLS, S.A. & McCARTY, K.S. SNR. (1983). Relationship of age and menopausal status to estrogen receptor content in primary carcinoma of the breast. *Ann. Surg.*, **197**, 123.

McTIERNAN, A., THOMAS, D.B., JOHNSON, L.K. & ROSEMAN, D. (1986). Risk factors for estrogen receptor-rich and estrogen receptor-poor breast cancers. *J. Natl Cancer Inst.*, **77**, 849.

MOHLA, S., SAMPSON, C.C., KHAN, T. & 6 others (1982). Estrogen and progesterone receptors in breast cancer in black Americans. *Cancer*, **50**, 552.

MOOLGAVKAR, S.H., DAY, N.E. & STEVENS, R.G. (1980). Two-stage model for carcinogenesis: Epidemiology of breast cancer in females. *J. Natl Cancer Inst.*, **65**, 559.

OSBORNE, M.P., ROSEN, P.P., LESSER, M.L. & 5 others (1983). The relationship between family history, exposure to exogenous hormones, and estrogen receptor protein in breast cancer. *Cancer*, **51**, 2134.

PAPATESTAS, A.E., PANVELINALLA, D., PERTSEMLIDIS, D., MULVIHILL, M. & AUFSESI, A. JNR. (1980). Association between estrogen receptors and weight in women with breast cancer. *J. Surg. Oncol.*, **13**, 177.

ROHAN, T.E. & BAIN, C.J. (1987). Diet in the etiology of breast cancer. *Epidemiol. Rev.*, **9**, 120.

ROHAN, T.E., RECORD, S.J. & COOK, M.G. (1987). Repeatability of estimates of nutrient and energy intake: The quantitative food frequency approach. *Nutr. Res.*, **7**, 125.

ROHAN, T.E., McMICHAEL, A.J. & BAGHURST, P.A. (1988). A population-based case-control study of diet and breast cancer in Australia. *Am. J. Epidemiol.* (in the press).

STANFORD, J.L., SZKLO, M. & BRINTON, L.A. (1986). Estrogen receptors and breast cancer. *Epidemiol. Rev.*, **8**, 42.

STANFORD, J.L., SZKLO, M., BORING, C.G. & 4 others (1987). A case-control study of breast cancer stratified by estrogen receptor status. *Am. J. Epidemiol.*, **125**, 184.

THOMAS, S. & CORDEN, M. (1970). Tables of composition of Australian foods. Australian Government Service: Canberra.

WALD, N. (1987). Retinol, beta-carotene and cancer. *Cancer Surv.*, **6**, 635.

WILLETT, W.C., SAMPSON, L., STAMPFER, M.J. & 5 others (1985). Reproducibility and validity of a semi-quantitative food frequency questionnaire. *Am. J. Epidemiol.*, **122**, 51.